# Risk Assessment of Cardiovascular Disease Among Adults Attending Primary Healthcare Centers in Riyadh City 2015

**DOI:** 10.7759/cureus.32503

**Published:** 2022-12-14

**Authors:** Bader Alibrahim, Jaber Sharaheeli, Lujain Alassaf, Eman E Abd-Ellatif

**Affiliations:** 1 Preventive Medicine, Field Epidemiology Training Program, Riyadh, SAU; 2 Population Health Management, Center for National Health Insurance, Riyadh, SAU; 3 Faculty of Medicine, Public Health & Preventive Medicine, Mansoura University, Mansoura, EGY

**Keywords:** cholesterol, smoking, obesity, hypertension, types 2 diabetes, cardio vascular disease

## Abstract

Background: The prevalence of cardiovascular disease (CVD) risk factors is expected to rise in Saudi Arabia as the prevalence of CVD risk factors rises. Effective primary CVD prevention necessitates risk assessment to categorize patients and select the most appropriate intervention for each category.

Objectives: To estimate the prevalence of CVD at primary healthcare (PHC) in Riyadh city and to categorize the at-risk population as a slow, intermediate, or high risk of CVD.

Methods: A cross-sectional study was carried out at seven PHC centers in Riyadh. Seven hundred participants (half of whom were males and the other half females) were selected at random from PHCs visitors, and data was collected using a structured questionnaire, as well as required measurements and laboratory investigations. The World Health Organization risk prediction charts were used to calculate CVD risk. Participants were assigned to one of three CVD risk categories (low, intermediate, high).

Results: Obesity was found to be the most common risk factor found in this study (53.2%). Overweight (31.2%), with females having higher BMI levels. The study found that 83.4% of participants had low CVD risk, 12.9% had intermediate risk, and 3.7% had high CVD risk category. Age, systolic blood pressure, cholesterol level, smoking, and prior diagnosis of diabetes were all statistically significant predictors of moderate and high CVD risk.

Conclusion: CVD risk factors were discovered to be common among study participants. It requires healthcare decision-makers to engage in community-based interventions to decrease the risk of CVD.

## Introduction

According to the World Health Organization, cardiovascular diseases (CVD) account for one in every three deaths worldwide. CVD accounts for nearly 17 million deaths in a year [1]. The disease burden is expected to rise by 17% per year until 2015 [2]. A thorough review of the literature and the experience of many countries confirms that CVD is preventable, so there is no reason for healthcare decision-makers not to make it a top priority for prevention and management. Countries’ experiences have shown that the more CVDs are controlled, the less money they spend on healthcare [3-5]. Therefore, community-based interventions by countries must be implemented to control this upraising disaster. To develop effective primary prevention strategies, we should focus on CVD risk factors.

There are many risk factors for CVD. Age, smoking, diabetes mellitus, high blood pressure, high serum total cholesterol, elevated serum low-density lipoprotein cholesterol (LDL-C), and low serum high-density lipoprotein cholesterol are all major and independent risk factors for CVD (HDL-C). The total risk of getting CVD in a person can be estimated by summing up all the associated major risk factors [6]. Obesity, physical inactivity, and a family history of CVD, as well as elevated serum triglycerides, elevated serum homocysteine, and inflammatory markers like C-reactive protein, are all risk factors. However, the American Heart Association considers obesity and physical inactivity to be major risk factors [7,8].

Such risk factors predict an escalation in the CVD burden in the Kingdom of Saudi Arabia (KSA) and an associated increase in mortalities and morbidities. According to the World Health Organization (WHO) report on disease mortality and burden, KSA has 55-95 per 100,000 mortalities due to stroke and 191-541 per 100,000 mortalities due to ischemic heart diseases [9]. There are, however, insufficient official, national, and generalizable studies to estimate the current situation and distribution of these cases. There is compelling evidence that CVD mortality and morbidity have decreased in countries where preventive measures [10]. As a result, the purpose of this study is to provide an overview of the CVD situation and risk factors in Riyadh’s primary healthcare centers. The findings of this study could aid in making informed decisions about non-communicable disease services and preventive measures in Riyadh.

The objective of this study is to estimate the prevalence of CVD risk factors and the percentage of people at risk for CVD in Riyadh’s PHC settings and to categorize the at-risk population as high, intermediate, or low risk of CVD using the WHO’s risk prediction chart.

## Materials and methods

Study design

The study is a cross-sectional analytical study.

Study population

The* *inclusion criteria included Saudi visitors of primary healthcare centers, males and females aged 18 or above, attending the PHC center designated for the research for any reason other than an emergency referral and providing consent to participate in the study. Pregnant women, people with clinical features suggestive of sudden cardiovascular events, such as a heart attack, angina, heart failure, arrhythmias, stroke, transient ischemic attack, and pre-existing cardiovascular disease (newly diagnosed or if not assessed in a specialist facility), were excluded from the study. These patients must be referred to be included in the study.

Study setting

The study was conducted at the Ministry of Health primary healthcare units in Riyadh, as the Saudi population of Riyadh is the primary user of Ministry of Health PHC centers. The majority of non-Saudis have private insurance and use other facilities for primary healthcare.

Sample size and type

The calculated sample size was 700. Sampling type was stratified random sampling with equal allocation.

Data collection

According to MOH allocation, Riyadh has 86 PHC centers spread across seven sectors. The participants were selected from seven PHC centers in the seven sectors. Each sector had one PHC center chosen at random, with a total of 100 participants from each center. Because there are not many patients visiting these PHC centers, and it is impractical to choose participants at random, all visitors to the PHC centers were invited to participate in this study. Half of the sample was collected from the male section and the other half from the female section.

The data was collected by physicians and nurses in the designated centers until the sample size requirement was met. Data was collected using a structured questionnaire in Arabic. The nurses completed one section of the questionnaire, the doctors completed another, and the lab technician completed the third.

The questionnaire was divided into three parts. The first part of the questionnaire was about demographic data (age, sex, educational level). The first part also included: Height of measurement, weight, and body mass index ("BMI"), measurement of waist circumference, and systolic blood pressure. The second part of the questionnaire included the previous history and the family history of CVD, diabetes mellitus, smoking, and physical activity. The third part of the questionnaire included the results of the blood sample for the tests of blood glucose, total cholesterol, HDL, and triglycerides. Laboratory investigations were held in the same PHC centers.

After finishing the data collection process, we classified the participants into various categories of risk of CVD by using a risk prediction chart. The WHO/International Society of Hypertension (ISH) risk prediction chart [11] was developed as a result of a WHO study to determine the disease burden. This chart has been used as a general predictor to indicate public health and clinical decisions in healthcare settings.

Data analysis

The information was entered centrally in Microsoft Excel (Redmond, USA) after adequate counterchecking and cleaning. The data were analyzed using IBM Corp. Released 2010. IBM SPSS Statistics for Windows, Version 19.0. Armonk, NY: IBM Corp. Mainly descriptive statistics were used in this study. A multinomial logistic model was used to determine the relationship between CVD risk levels and risk factors. The p-value < 0.05 was considered statistically significant.

Ethical considerations

All PHC attendees were informed about the research and its potential benefits to their health. The participant provided consent which was confirmed by another healthcare professional. The team provided no incentives to the participants. The IRB approval was obtained from the College of Medicine, King Saud University.

## Results

In this study, 700 people with a mean age of 42.6 were recruited. The risk of CVD was observed to be low across the majority of the sample (83.4%). The intermediate CVD risk was 12.9%, and the high risk was 3.7%. The risk of CVD was higher among participants aged 50 and above.

In terms of physical activity, approximately 36% reported being physically inactive, while only about 8% were moderately or highly active. Furthermore, more than half of the participants (53.2%) were obese, and (31.2%) were overweight (Table 1), with females having higher BMI levels.

**Table 1:** Descriptive statistics of participants' demographics and medical history

Variable		N (%)
Gender	Male	349 (49.9)
	Female	351 (50.1)
Education	No education	98 (14.3)
	Primary	85 (12.4)
	Secondary	101 (14.7)
	University	223 (32.5)
	Higher Education	180 (26.2)
BMI	Underweight	11 (1.6)
	Normal weight	97 (14)
	Overweight	216 (31.2)
	Obese	368 (53.2)
Level of physical activity	No activity	158 (22.6)
	Low	251 (35.9)
	Moderate	237 (33.9)
	High	54 (7.7)
Family history of CVD	Yes	284 (40.6)
	No	416 (59.4)
Diagnosed with Type II diabetes	Yes	106 (15.1)
	No	594 (84.9)
Smoking	Yes	130 (18.6)
	No	570 (81.4)
Risk category	Low	5208 (83.4)
	Moderate	1428 (12.9)
	High	1158 (3.7)

In terms of medical history, 15.1% of participants had previously been diagnosed with type II diabetes, and more than 40% had a relative who had a CVD event (Table 1). Furthermore, males had higher average blood glucose and cholesterol levels than females, as well as higher scores for moderate and high CVD risk (Table 2).

**Table 2:** Participants' characteristics by gender

Variable	Category	Male N (%)*	Female N (%)*
Risk category	Low	271 (46.4)	313 (53.6)
	Medium	59 (65.6)	31 (34.4)
	High	19 (73.1)	7 (26.9)
BMI	Underweight	6 (54.5%)	4 (45.5%)
	Normal weight	52 (53.6%)	45 (46.4%)
	Overweight	133 (61.6%)	82 (38.4%)
	Obese	156 (42.4%)	212 (57.6%)
Level of physical activity	No activity	103 (65.2)	55 (34.8)
	Low	108 (43.0)	143 (57.0)
	Moderate	119 (50.2)	118 (49.8)
	High	19 (35.2)	35 (64.8)
Education level	No education	26 (26.5)	72 (73.5)
	Primary	42 (49.4)	43 (50.6)
	Secondary	58 (57.4)	43 (42.6)
	University	137 (61.4)	86 (38.6)
	Higher education	78 (43.3)	102 (56.7)
Smoking	Yes	115 (88.5)	15 (11.5)
	No	234 (41.1)	336 (58.9)
Family history of CVD	Yes	140 (49.3)	144 (50.7)
	No	209 (51.9)	194 (48.1)
Diagnosed with Type II diabetes	Yes	61 (57.5)	45 (42.5)
	No	288 (48.5)	306 (51.5)

The following factors (Table 3) were linked to a moderate risk of CVD: Age ([OR = 1.341; 95% CI: 1.235-1.457], p< .001), high systolic blood pressure ([OR = 1.262; 95% CI: 1.181-1.348], p< .001), smoking ([OR = 1.980; 95% CI: 1.188-3.300], p< .001) and a prior diagnosis of diabetes ([OR = 2.306; 95% CI: 1.828-2.973), p< .001). The factors that were statistically significant for moderate risk were also statistically significant for high CVD risk. Furthermore, stronger associations were observed for each of the predictors of high risk when compared to the reference category “low.”

**Table 3:** Association between participant’s characteristics and the risk of CVD

		Moderate				High			
Variable		OR	95% CI		P-value	OR	95% CI		P-value
Age		1.341	1.235	1.457	.001	1.852	1.555	2.206	.001
Systolic blood pressure		1.262	1.181	1.348	.001	1.621	1.398	1.879	.001
Blood glucose level		1.008	1.000	1.017	.062	1.004	.990	1.018	.593
Cholesterol level		1.013	1.001	1.025	.054	1.021	1.000	1.042	.068
Gender		1.031	.353	3.011	.956	1.759	.263	11.768	.560
Level of physical activity	No activity	.720	.190	2.724	.628	.841	.099	7.134	.874
	Low	.650	.207	2.046	.462	.176	.017	1.801	.143
	Moderate or High	1	.	.	.	1	.	.	.
Smoking		1.980	1.188	3.300	.001	3.217	1.417	7.305	.001
previously diagnosed with Type II diabetes		2.306	1.828	2.973	.001	3.012	2.102	3.982	.001

## Discussion

Increasing age, high systolic blood pressure, diabetes diagnosis, and smoking were all linked to an increased risk of CVD. These risk factors are known to be major and independent risk factors of CVD [6]. Most participants in this study are at low risk of CVD, with only 3.7% at high risk. 12.9% of the participants have an intermediate risk. However, some risk factors were not considered in the risk calculation, and we can expect more people to be at risk than this study indicates.

As far as we know, this is the first study to use risk prediction charts to assess 10-year CVD risk among visitors to Riyadh’s PHC centers. A similar study was conducted in Jeddah (King AbdulAziz University) in 2014 among medical students to assess the 30-year risk for CVD using the Framingham risk score. The results for intermediate and high risk were 2.3% and 0.5%, respectively [12]. The percentages in the Jeddah study are lower than the percentages in our study, which may be due to the age of the participants (young age) and the smaller sample size (214 medical students) in the Jeddah study.

A 2004 study of U.S. adults to predict the 10-year risk of CVD found that 81.7% had a low risk, 15.5% had an intermediate risk, and 2.9% had a high risk [13]. The study included participants aged 20-79, and the risk increased with age and was higher in men than in women. Even though the study among U.S. adults had a much larger sample size, the results are nearly identical to ours (13,769 participants representing more than 157 million U.S. adults).

The risk of CVD increases with advancing age. This is the result of the atherosclerosis cumulative effect. Advancing age comes with accumulating amounts of coronary atherosclerosis. This increased plaque burden increases the risk of coronary events [14]. Furthermore, growing older is often associated with other major risk factors, such as hypertension and diabetes. This coexistence of the major risk factors increases the risk of CVD.

Both type 1 and type 2 diabetes mellitus are considered risk factors for CVD. Type 2 diabetes is common, and it is especially concerning because it typically affects the elderly and coexists with other major risk factors. Diabetes has been reported as a common disease in Saudi Arabia in numerous studies, and this study revealed that 15.1% of the participants were diabetics. This result is consistent with all other studies.

Diabetes mellitus was published in 2004 with a prevalence of 23.7% [15]. Diabetes was found in 14.8% of males and 11.7% of females in the MOH national survey of 2013 [16], and the percentage in our study is very close to that. Only some older studies showed a lower prevalence of diabetes than our study. According to Elhazmi [17], the overall prevalence of diabetes mellitus in Riyadh was 4.76% in males and 4.10% in females in 1995.

Hypertension is a major risk factor for CVD. Its coexistence with the other risk factors increases the risk of CVD. According to the MOH national survey of 2013 [16], the prevalence of hypertension was higher among those aged 65 and above (65.2%). Hypertension is 3.5 times more among diabetics (38.9%) compared to non-diabetics (11.9%). The prevalence of 24.1% in our study is consistent with the prevalence of 24.1% in the Alnozha 2007 nationwide study [18].

Smoking increases the risk of CVD. The prevalence of smoking in our study, 18.6%, is consistent with a 1999 study on the prevalence of smoking in three Saudi Arabian regions [19]. The overall prevalence of current smoking in that study was 21.1% for males and 0.9% for females. A study conducted at King Faisal University in Dammam, published in 2007, found that 19% of male students were current smokers [20]. According to a 2011 study conducted among university students at King Saud University in Riyadh, the prevalence of smoking among students was 14.5% [21]. Another study, also published in 2011, among medical students in Saudi Arabia’s western region, found that smoking was prevalent in (24.8%) percent of males and (9.1%) percent of females [22]. The prevalence of smoking in previous studies was approximate to the prevalence of smoking in this study. The prevalence of smoking in the MOH national survey of 2013 was 11.2% [16], which is lower than the prevalence in our study.

A very low prevalence of smoking (all kinds of smoking) is seen among females (including our study prevalence) in Saudi Arabia. We must consider the social norms in Saudi Arabia and the Arab world that prevent women from telling the truth about this issue. We should also note that recent studies have begun to focus on hookah smoking, which is becoming an acceptable habit for both men and women in Saudi and Arab societies.

Obesity raises blood pressure and predisposes to type 2 diabetes, as well as increasing the risk of CVD. The high prevalence of obesity in this study (52%) is higher than in most Saudi studies. The MOH national survey of 2013 showed the prevalence of obesity at 28.7% only [16]. After examining 17,232 Saudi subjects, Alnozha’s nationwide study of obesity in Saudi Arabia, published in 2005, found an overall prevalence of overweight (36.9%) and a prevalence of obseity (35.5%) [23]. Obesity was found to be 16% among males and 24% among females in a national survey of diabetes, obesity, and hypercholesterolemia in Saudi Arabia published in 1995 by Al-Nuaim [24]. An old study conducted in Al-Thuqbah primary healthcare center (eastern province of KSA) in 1998 revealed a prevalence of obesity of 37.9% and a prevalence of overweight of 33% [25]. In an a 1992 study of 300 diabetic Saudi women in Saudi Arabia’s eastern province, 57% were found to be obese [26]. Nonetheless, it appears that the obesity situation is not improving.

Physical inactivity increases the risk of CVD and harms the major risk factors of CVD. Our study’s prevalence of physical inactivity (58.5%) is consistent with the 2013 MOH national survey prevalence, which was 60% [16]. Previous studies done in the kingdom about physical inactivity showed high prevalence of inactivity. In a national study on physical activity among Saudi males and females aged 30-70, published in 2007 by Alnozha, the prevalence of physical inactivity was very high (96.1%) [27]. Physical inactivity also was 68.3% in the study of Al-Thuqbah primary healthcare center [25].

In general, the prevalence of the major CVD risk factors that are statistically correlated with CVD risk in this study is consistent with other studies among Saudi populations in different regions, as well as national surveys. Some risk factors in this study have a higher prevalence than in other Saudi studies and surveys.

Recommendations

The following are the recommendations for preventing cardiovascular disease in people who have cardiovascular risk factors (according to the participant risk category):

Low-risk category: Low-risk category does not mean “No” risk. As a result, recommendations and health education for leading a healthy lifestyle should be made available.

Intermediate risk category: Participants in this category are at a moderate risk to develop fatal or nonfatal CVD within 10 years. Individuals in this category should be monitored to check their risk level every 6-12 months and advised on how to improve their lifestyle.

High-risk category: Monitoring should take place every 3-6 months, with an emphasis on improving one’s lifestyle and referral to a higher level of healthcare for preventive medical intervention if necessary.

Limitations

Some issues affected the accuracy of our assessment of the CVD risk. The severity of the risk factors is not taken into account in the risk prediction chart (e.g., severe hypertension or heavy smoking). The risk prediction charts have limitations when applied to non-western people, and they may underestimate the risk. The risk chart does not include some of the CVD risk factors. Now and then, there is a debate about including other risk factors such as C-reactive protein, fibrinogen, and waist-hip ratio. So far, such debates have not resulted in additional recommendations or changes to the scoring systems.

Some of the factors in this study, such as smoking and physical activity, are based on participant self-reporting, which understates the true prevalence of these factors and, thus, the risk of CVD.

## Conclusions

The prevalence of CVD risk factors is increasing with time, and it mandates community-based interventions by healthcare decision-makers. Having nearly identical, or even worse, risk factors numbers after two decades necessitate a review of current health promotion activities and an urgent need to investigate more effective measures. More efforts are needed to promote healthy attitudes and behaviors related to Saudi people’s lifestyles to reduce the risk of CVD.
